# Progressive Multifocal Leukoencephalopathy in a 62-Year-Old Immunocompetent Woman

**DOI:** 10.1155/2014/549271

**Published:** 2014-02-23

**Authors:** Venkata C. Gourineni, Tristan Juvet, Yogesh Kumar, Doru Bordea, Kanaga N. Sena

**Affiliations:** ^1^Department of Internal Medicine, Bridgeport Hospital, Yale New Haven Health, 267 Grant Street, Bridgeport, CT 06610, USA; ^2^Department of Radiology, Bridgeport Hospital, Yale New Haven Health, 267 Grant Street, Bridgeport, CT 06610, USA

## Abstract

Progressive multifocal encephalopathy (PML) is a rare demyelinating disease that typically presents in immunodeficient patients. We report a case of a previously healthy 62-Year-Old woman who suffered from an unsteady gait, throbbing headaches, and progressive left-sided weakness and numbness. Stroke was initially suspected based on imaging and symptoms. A series of follow-up magnetic resonance images of the brain showed a right parietal lesion growing in size as the patient became unable to walk and experienced increasing lethargy and confusion. A biopsy of the lesion was positive for the John Cunningham virus (JCV). A diagnosis of PML was made and she was started on mefloquine. No improvement was seen on this treatment and her condition worsened. Although PML remains uncommon in immunocompetent individuals, it cannot be ruled out based on their immune status. Although the exact cause remains uncertain, underlying or transient states of immunosuppression may be responsible for reactivation of the JCV in these patients.

## 1. Introduction

Progressive multifocal leukoencephalopathy (PML) is a demyelinating disease of the central nervous system that typically occurs in immunosuppressed individuals. It is caused by reactivation of the John Cunningham virus (JCV) and infection of glial cells. It is often fatal, with a median life expectancy of less than six months following onset of symptoms [1]. Reports of PML afflicting immunocompetent patients are extremely rare but not unknown. We describe a patient with no previous medical issues and an intact immune system who presented with PML.

## 2. Case Presentation

A 62-year-old female with no past medical history presented to the emergency department after a one-month history of progressive left-sided numbness, weakness, and unsteady gait. She had also experienced multiple falls and bladder incontinence over the past two weeks. She complained of frequent throbbing headaches in the occipital area that were not relieved by analgesics. The patient denied fever, chills, confusion, visual changes, or seizures. She had not sought any medical attention prior to this point and was not on any medication. She was of Portuguese origin and was married with two adult children. She had previously worked as a salesperson. She had never smoked and had no history of illicit drug or alcohol use. She denied recent travel, sick contacts, or exposure to wildlife. Family history was unremarkable.

On examination, she was alert and oriented to person, time, and place. Some slight left-sided tongue deviation as well as decreased motor strength in the left upper and lower extremities was noted, including a mild drift of the left arm. Deep tendon reflexes were found to be normal. She had diminished light touch on the left side. Pupils were equal and reactive and extraocular movements were fully intact. Blood pressure was 145/75 mm Hg. Her lab work on admission was found to be normal, including a white blood cell count of 9000 cells/*μ*L. Her electrocardiogram showed normal sinus rhythm. Computed tomography (CT) scan of the head outlined an area of diminished attenuation in the high right parietal lobe that was suspicious for edema. No midline shift or mass lesions were noted.

**Figure 1:**
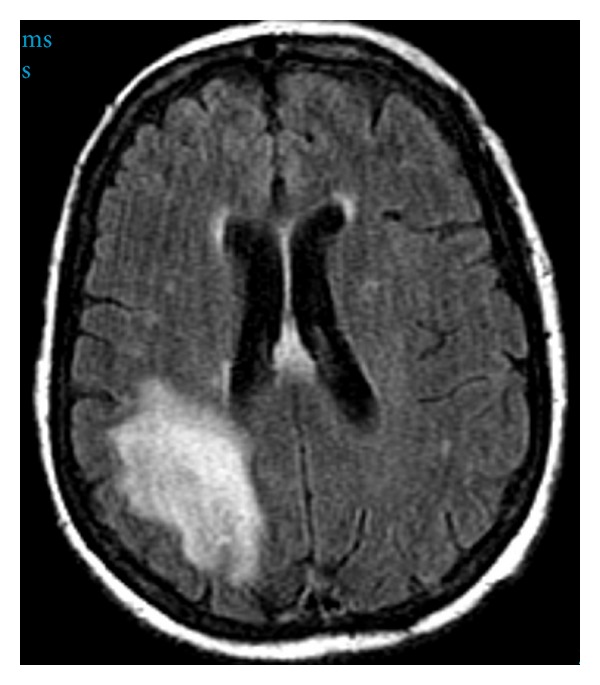
Axial T2-FLAIR image shows increase in signal intensity in the subcortical white matter involving the U-fibers in the right parietal lobe.

Magnetic resonance imaging (MRI) was performed to better characterize the CT findings (Figure 1). Multiple abnormal areas were seen throughout the periventricular and subcortical white matter of the bilateral cerebral hemispheres, including the right parietal lobe region. No enhancement was noted in these areas, making malignancy less likely. The ventricles were normal in size and configuration. Carotid Doppler ultrasounds did not show significant stenosis. A transesophageal echocardiogram showed a normal ejection fraction and no thrombi, with the presence of a patent foramen ovale with a right-to-left shunt. The patient's condition did not show any improvement over the following five days and a follow-up brain MRI performed at this time showed decreased perfusion of the right parietal area in comparison to the contralateral side. Based on her CT scan, MRI, echocardiogram, and neurological exam, acute on superimposed chronic infarcts was suspected and the patient was transferred to the inpatient rehabilitation unit ten days following admission and placed on an antiplatelet agent.

During rehabilitation, her condition worsened. At day 15 of her hospitalization, she became unable to walk due to worsening weakness of her left lower extremity and experienced high fever. She also complained of occasional vertigo. Her thinking became disorganized with diminished attention. No rigidity, myoclonus, or cogwheeling was noted. The patient's leukocyte count was elevated to 18,900 cells/*μ*L, with 67% lymphocytes. Urine and blood cultures returned negative. Anti-nuclear antibodies and rheumatoid factor were negative. HIV status was negative on ELISA and polymerase chain reaction (PCR) testing. A CT of the abdomen and chest revealed only mild hepatosplenomegaly. Follow-up brain MRI showed a 0.5 centimeter increase in the diameter of the right parietal lesion.

Due to lack of clear diagnosis, biopsy of the right parietal lesion was performed, in addition to analysis of the cerebrospinal fluid (CSF). This showed demyelinating macrophages in addition to enlarged bizarre-shaped cells with nuclear inclusions that stained positive for antibodies against simian virus 40 (SV40). SV40 immunochemistry is known to cross-react with the JCV [2]. A diagnosis of PML was made based upon these findings. This was confirmed by positive PCR and *in situ *hybridization results for JCV from CSF samples were sent to the National Institute of Health. She was started on a mefloquine trial, which had previously shown some success in inhibiting JCV replication. Unfortunately, she did not show any improvement while on mefloquine and continued experiencing a decline in mental function over the following four months. She ultimately became comatose and died.

## 3. Discussion

It has been recognized that the JCV is highly prevalent in the adult population, with 50–90% of healthy individuals having been exposed to the virus [3]. Approximately 85% of the population has antibodies to JCV. The virus' purported site of latency in the human body is currently under debate. It is known that it not only establishes itself in the kidney and bone marrow but also may be present in the brain prior to reactivation [4]. Currently, about 79% of individuals afflicted with PML have AIDS, 13% have hematological malignancies, and 5% are organ transplant recipients [5]. Recently, PML has also been associated with the use of newer immunomodulating medication such as natalizumab [6, 7], rituximab [8], and efalizumab, representing 3% of cases [3].

Cases of PML in seemingly immunocompetent individuals are very uncommon. The exact cause of JCV reactivation in patients who are not immunodeficient remains controversial. A series of cases by Gheuens et al. showed that a certain degree of mild immunosuppression was present in 38 cases of individuals with PML who were HIV-negative and free of malignancies. The associated conditions among this subset of patients were variable and included hepatic cirrhosis, chronic renal failure, dermatomyositis, pregnancy, and Alzheimer's disease, none of which were present in our patient [9]. In addition, three patients were clinically suspected of having a degenerative disease. A case report from Tan et al. described a healthy patient with a CD4+ count of 1200 cells/*μ*L who was diagnosed with PML and recovered following a six-month mefloquine treatment [10]. The role of mefloquine in the patient's recovery remains debatable as the clinical trial was recently terminated after failing to show improvement in subjects [10–12].

A case reported by Naess et al. was of a previously healthy 35-year-old male with a CD4+ count of 994 cells/*μ*L who was diagnosed with PML by brain biopsy [13]. He was treated with intravenous cidofovir and showed clinical improvement as well as regression of white matter lesions on MRI. Despite his treatment, it is suspected that his recovery was spontaneous. Cidofovir, along with mefloquine, cytosine arabinoside, and interferon alpha were not associated with any survival benefits in patients with PML, despite some *in vitro* efficacy against JCV [3]. It appears that the body's ability to mount a strong immune response to the JCV virus can result in disappearance of the disease [14]. This was shown in HIV-positive patients, where initiation of highly active antiretroviral therapy (HAART) was associated with the best prognosis [15].

It has been postulated that a transient dysfunction of the immune system caused by a subclinical viral infection may be responsible for reactivation of JCV within the setting of an immunocompetent individual [10]. However, there are no proven cases of this occurring and we do not have any reason to suspect this in our patient. Another possibility is idiopathic CD4+ lymphocytopenia, a rare condition that is defined as a documented CD4+ cell count of less than 300 cells/*μ*L in HIV-negative patients. A recent review of the initial presentation of patients with idiopathic CD4+ lymphocytopenia by Zonios et al. described one case of PML among 39 individuals [16]. A T-cell subset count was not obtained in our particular case. However, complete lymphocyte counts were constantly found to be normal. Idiopathic CD4+ lymphocytopenia will usually present with absolute lymphocytopenia, making it an unlikely condition in our patient's case.

The patient's clinical course, radiographic findings, and histology were highly typical of PML, despite showing no signs of depressed immune function. The progression of the disease in this patient is unique, considering that previous immunocompetent cases with PML reportedly recovered following hospitalization. Although the effectiveness of pharmacological treatment has not been proven, it did not seem to alter the course of the disease in our patient. The cause of viral reactivation in her case remains unknown. The possibility of an undiagnosed degenerative disease cannot be excluded in her case although her younger age would make this less likely. PML may present in immunocompetent individuals although controversy remains as to whether a certain degree of immunosuppression, either transient or chronic, is required for this occurrence.

## Abbreviations

PML: Progressive multifocal leukoencephalopathy
JCV: John Cunningham virus
CT: Computed tomography
MRI: Magnetic resonance imaging
HIV: Human immunodeficiency virus
PCR: Polymerase chain reaction
ELISA: Enzyme-linked immunosorbent assay
SV40: Simian virus 40
AIDS: Acquired immune deficiency syndrome
HAART: Highly active antiretroviral therapy.
